# The anti-epithelial cell adhesion molecule (EpCAM) monoclonal antibody EpMab-16 exerts antitumor activity in a mouse model of colorectal adenocarcinoma

**DOI:** 10.3892/ol.2020.12246

**Published:** 2020-10-23

**Authors:** Hideki Hosono, Tomokazu Ohishi, Junko Takei, Teizo Asano, Yusuke Sayama, Manabu Kawada, Mika K. Kaneko, Yukinari Kato

**Affiliations:** 1 Department of Antibody Drug Development, Tohoku University Graduate School of Medicine, Sendai, Miyagi 980-8575, Japan; 2 Institute of Microbial Chemistry (BIKAKEN), Numazu, Microbial Chemistry Research Foundation, Numazu, Shizuoka 410-0301, Japan; 3 New Industry Creation Hatchery Center, Tohoku University, Sendai, Miyagi 980-8575, Japan

**Keywords:** EpCAM, monoclonal antibody, ADCC, CDC, antitumor activity, colorectal adenocarcinoma

## Abstract

The epithelial cell adhesion molecule (EpCAM), which is a calcium-independent homophilic intercellular adhesion factor, contributes to cell signaling, differentiation, proliferation and migration. EpCAM is essential for carcinogenesis in numerous types of human cancer. The purpose of the present study was to establish an anti-EpCAM monoclonal antibody (mAb) for targeting colorectal adenocarcinomas. Thus, an anti-EpCAM mAb, EpMab-16 (IgG_2a_, κ), was established by immunizing mice with EpCAM-overexpressing CHO-K1 cells, and validated using flow cytometry, western blot, and immunohistochemical analyses. EpMab-16 reacted with endogenous EpCAM specifically in a colorectal adenocarcinoma cell line as determined by flow cytometry and western blot analyses. Immunohistochemical analysis demonstrated that EpMab-16 stained a plasma membrane-like pattern in clinical colorectal adenocarcinoma tissues. The dissociation constant (*K*_D_) for EpMab-16 in a Caco-2 colorectal adenocarcinoma cell line determined by flow cytometry was 1.8×10^−8^ M, suggesting moderate binding affinity of EpMab-16 for EpCAM. Whether the EpMab-16 induced antibody-dependent cellular cytotoxicity (ADCC) and complement-dependent cytotoxicity (CDC) against Caco-2 or antitumor activity was then assessed in a murine xenograft model. *In vitro* experiments revealed strong ADCC and CDC induction in Caco-2 cells by EpMab-16 treatment. *In vivo* experiments in a Caco-2 ×enograft model demonstrated that EpMab-16 treatment significantly reduced tumor growth compared with that in mice treated with the control mouse IgG. These results suggested that EpMab-16 may be a promising treatment option for EpCAM-expressing colorectal adenocarcinomas.

## Introduction

Cellular junctions comprise a range of cell adhesion molecules (CAM) and are essential for maintaining tissue architecture (1). The four major CAM families are integrins, cadherins, selectins and the immunoglobulin CAM superfamily (2). Integrins are composed of two or more noncovalently-associated membrane-spanning subunits α and β (3). The specific combination of α and β subunits confers specificity for various extracellular ligands and their respective intracellular signaling events, and each combination of α and β represents a significant receptor family within the context of interaction with the extracellular matrix (3). Cadherins are calcium-dependent glycoproteins, which possess an extracellular CAM domain with three to five internal repeats, a single-span transmembrane domain and an intracellular domain (2). The extracellular domain of selectins consists of a calcium-dependent lectin domain and an epidermal growth factor (EGF)-like domain (2). Selectins also contain a hydrophobic transmembrane domain and a short cytoplasmic tail (2). The Ig-CAMs are calcium-independent, with an extracellular domain comprising a ligand-binding region of four to six Ig-like repeats, one to five fibronectin-like repeats, a transmembrane domain and an intracellular component (1). Although these families are predominant, a number of CAMs do not share any structural similarities with them, such as the epithelial cell adhesion molecule (EpCAM) (4).

EpCAM is one of the first identified human tumor-associated biomarkers (5) and is now considered to be a marker of tumor-initiating cells (6). EpCAM is a transmembrane, calcium-independent, homophilic, intercellular adhesion glycoprotein (314 amino acids; 40 kDa) with three distinct domains: An extracellular domain (EpEX, 265 amino acids), a transmembrane domain and an intracellular domain (EpICD, 26 amino acids) (7). The cleaved EpICD enters the nucleus, leading to the activation of the β-catenin/c-Myc signaling pathway to promote cancer cell proliferation (8). EpCAM functions include cell signaling, differentiation and migration in addition to adhesion and proliferation (4). EpCAM has been implicated in carcinogenesis and is expressed robustly in various types of human epithelial cancers, such as lung, breast, ovarian, cervical and colorectal cancer (CRC), suggesting that it may be a promising target for cancer diagnosis and therapy (9–11).

According to GLOBOCAN 2018 data, CRC is the third most commonly occurring cancer and second leading cause of cancer-associated death in the world, with ~881,000 deaths estimated for 2018 (12). Although surgical removal of cancer followed by adjuvant therapy is one of the most effective treatments, recurrences are inevitable (13–15). Antibody-based treatments are also currently used in patients with advanced CRC; however, the prognosis and clinical outcomes of patients with CRC remain poor (16). Therefore, new strategies are required to improve the effectiveness of CRC treatment.

The present study developed an anti-EpCAM monoclonal antibody (mAb) using cell-based immunization and screening (CBIS) methods (17) aiming to determine whether these anti-EpCAM mAbs induced antibody-dependent cellular cytotoxicity (ADCC), complement-dependent cytotoxicity (CDC) or antitumor activity against CRC in a murine xenograft model.

## Materials and methods

### 

#### Antibodies

Purified mouse IgG (cat. no. I8765) and mouse IgG_2a_ (cat. no. M7769) were purchased from Sigma-Aldrich; Merck KGaA. Anti-EpCAM mAbs were purified using Protein G-Sepharose (Cytiva).

#### Animals

All animal experiments were performed in accordance with institutional guidelines and regulations to minimize animal suffering and distress in the laboratory. The Institutional Committee for Experiments of the Institute of Microbial Chemistry (Numazu, Japan) approved the animal studies for ADCC and antitumor activity (approval no. 2019-066). Mice were monitored for health and weight every 1–5 days. Experiments on mice were conducted in ≤3 weeks. Weight loss >25% or tumor size >3,000 mm^3^ were identified as humane endpoints for euthanasia. At humane and experimental endpoints, mice were euthanized by cervical dislocation, and death was verified by validating respiratory and cardiac arrest.

#### Cell lines

P3X63Ag8U.1 (P3U1), CHO-K1 and Caco-2 cells were obtained from the American Type Culture Collection. The Genome Network Project clone IRAK021G03 (EpCAM) was provided by the RIKEN BioResource Research Center through the National BioResource Project of the MEXT and AMED agencies of Japan (18–21). EpCAM DNA plus a C-terminal PA tag recognized by the anti-PA tag mAb (NZ-1) was subcloned into a pCAG-Ble vector (FUJIFILM Wako Pure Chemical Corporation). CHO/EpCAM was established by transfecting pCAG/EpCAM-PA into CHO-K1 cells using the Neon Transfection System (Thermo Fisher Scientific, Inc.). CHO-K1 cells (1.5×10^6^) were transfected with 10 µg of plasmid DNA using 100 µl Neon tip, at room temperature. After 4 days, cells were incubated with 1 µg/ml anti-EpCAM mAb (clone 9C4; cat. no. 324202; BioLegend, Inc.) for 30 min on ice and subsequently with Alexa Fluor 488-conjugated anti-mouse IgG (1:1,000; cat. no. 4408; Cell Signaling Technology, Inc.) for 30 min on ice. Positive cells for anti-EpCAM mAb were sorted using an SH800 cell sorter (Sony Corporation), and stable transfectants were cultivated in RPMI-1640 medium (Nacalai Tesque, Inc.) containing 0.5 mg/ml zeocin (InvivoGen). Using TruGuide gRNA tool, gRNA of EpCAM (NM_002354) was selected from GeneArt predesigned gRNAs database (Thermo Fisher Scientific, Inc.). gRNA sequence used was GATCCTGACTGCGATGAGAG(cgg), which targeted exon 3 of EpCAM (Assay ID, CRISPR701274). Double strand gRNA sequence was subcloned into GeneArt CRISPR Nuclease Vector with OFP Reporter (Thermo Fisher Scientific, Inc.). Caco-2/EpCAM-knockout (BINDS-16) cells were generated by transfecting 10 μg of CRISPR/Cas9 plasmids for EpCAM (Thermo Fisher Scientific, Inc.) into Caco-2 cells (1.5×10^6^) for 7 days using a Neon transfection system with 100 µl Neon tip. Stable transfectants were established by cell sorting as aforementioned. P3U1, CHO-K1 and CHO/EpCAM cells were cultured in RPMI-1640 medium (Nacalai Tesque, Inc.). Caco-2 and BINDS-16 were cultured in Dulbecco's modified Eagle's medium (DMEM; Nacalai Tesque, Inc.). The medium was supplemented with 10% heat-inactivated fetal bovine serum (FBS; Thermo Fisher Scientific Inc.), 100 U/ml penicillin, 100 µg/ml streptomycin and 0.25 µg/ml amphotericin B (Nacalai Tesque, Inc.), and the cells were incubated at 37°C in a humidified atmosphere containing 5% CO_2_.

#### Hybridoma production

The CBIS method (17) was used in the present study to develop mAbs against EpCAM. Briefly, one BALB/c mouse was intraperitoneally (i.p.) immunized with CHO/EpCAM cells (1×10^8^ cells/500 µl) with Imject Alum adjuvant (Thermo Fisher Scientific Inc.). The procedure included three additional immunizations, followed by a final booster injection administered i.p. 2 days before spleen cell harvesting. Spleen cells (1×10^8^ cells) were then fused with mouse plasma cell myeloma P3U1 cells (1×10^7^ cells) using PEG1500 (Roche Diagnostics). The hybridomas were cultured in RPMI-1640 medium supplemented with hypoxanthine, aminopterin and thymidine for selection (50X solution; Thermo Fisher Scientific Inc.). Cell culture supernatants of hybridomas in each well of 96-well plates were mixed with 1×10^5^ CHO/EpCAM cells and were directly screened using flow cytometry.

#### Flow cytometry

Caco-2 cells (2.5×10^5^ cells/ml) were harvested after brief exposure to 0.25% trypsin in 1 mM ethylenediaminetetraacetic acid (EDTA; Nacalai Tesque, Inc.). Following washing with 0.1% bovine serum albumin (BSA; Nacalai Tesque, Inc.) in phosphate-buffered saline (PBS), Caco-2 cells were treated with 1 µg/ml of anti-EpCAM mAbs for 30 min at 4°C, followed by Alexa Fluor 488-conjugated anti-mouse IgG (1:1,000; cat. no. 4408; Cell Signaling Technology, Inc.). Fluorescence data were obtained using an EC800 Cell Analyzer (Sony Corporation), and analyzed using the originally installed EC800 software v1.3.6 (Sony Corporation).

#### Western blot analysis

Cell pellets were lysed in PBS with 1% Triton X-100 and 50 µg/ml aprotinin (cat. no. 03346-84; Nacalai Tesque, Inc.). Protein concentration was determined using the BCA assay. Cell lysates of CHO-K1, CHO/EpCAM, Caco-2 and BINDS-16 cells were boiled in sodium dodecyl sulfate sample buffer (Nacalai Tesque, Inc.). The samples (10 µg/lane) were then electrophoresed on 5–20% polyacrylamide gels (Nacalai Tesque, Inc.) and transferred to polyvinylidene difluoride membranes (Merck KGaA). Following blocking with 4% milk (Nacalai Tesque, Inc.) for 1 h, the membrane was incubated with anti-EpCAM (1 µg/ml) or anti-β-actin (1 µg/ml; clone AC-15; cat no. A5441; Sigma-Aldrich; Merck KGaA) antibodies for 1 h, followed by incubation with HRP-conjugated anti-mouse IgG (cat. no. P0260, Agilent Technologies, Inc.) or anti-rat IgG (cat. no. A9542; Sigma-Aldrich; Merck KGaA) at a 1:2,000 dilution for 1 h at room temperature. The membrane was developed using the ImmunoStar LD Chemiluminescence Reagent (FUJIFILM Wako Pure Chemical Corporation) and a Sayaca-Imager (DRC Co., Ltd.). All western blotting procedures were performed at room temperature.

#### Immunohistochemical analyses

Histological sections (4 µm) of a colorectal adenocarcinoma tissue array (cat. no. CO243b; US Biomax Inc.) were autoclaved directly in citrate buffer (pH 6.0; Nichirei Bioscience, Inc.) for 20 min. The sections were then incubated with 1 μg/ml anti-EpCAM mAb for 1 h at room temperature and treated using an Envision+ kit (Agilent Technologies, Inc.) for 30 min at room temperature. The color was developed using 3,3′-diaminobenzidine tetrahydrochloride (Agilent Technologies Inc.) for 2 min at room temperature, and sections were then counterstained with hematoxylin (FUJIFILM Wako Pure Chemical Corporation) for 2 min at room temperature. Hematoxylin and eosin (H&E) staining (FUJIFILM Wako Pure Chemical Corporation) was performed using consecutive colorectal adenocarcinoma tissue sections for 2 min at room temperature.

#### Determination of the binding affinity

Caco-2 cells were suspended in 100 µl serially diluted anti-EpCAM mAb (0.006–100 µg/ml), followed by the addition of Alexa Fluor 488-conjugated anti-mouse IgG (1:200; cat. no. 4408; Cell Signaling Technology, Inc.). Fluorescence data were obtained using an EC800 Cell Analyzer, and analyzed using the originally installed EC800 v1.3.6 software (Sony Corporation). The dissociation constant (*K*_D_) was calculated by fitting binding isotherms to built-in, one-site binding models in GraphPad Prism 6 (GraphPad Software, Inc.).

#### ADCC

ADCC induction by EpCAM was assayed as follows: A total of 6 female 5-week-old BALB/c nude mice were purchased from Charles River Laboratories, Inc. Following euthanasia by cervical dislocation, the spleens were removed aseptically, and single-cell suspensions were obtained by forcing spleen tissues through a sterile cell strainer (cat. no. 352360; Corning, Inc.) with a syringe. Erythrocytes were lysed by 10-sec exposure to ice-cold distilled water. The splenocytes were washed with DMEM and resuspended in DMEM with 10% FBS; this yield was designated as effector cells. Caco-2 cells were labeled with 10 µg/ml Calcein-AM (Thermo Fisher Scientific, Inc.) and resuspended in DMEM with 10% FBS. Caco-2 cells were transferred to 96-well plates at 2×10^4^ cells/well and mixed with the effector cells at an effector-to-target ratio of 50:1, along with 100 µg/ml anti-EpCAM mAb or control mouse IgG_2a_. Following a 4-h incubation, Calcein-AM release into the supernatant was measured in each well using a Power Scan HT microplate reader (BioTek Instruments, Inc.) with an excitation wavelength of 485 nm and an emission wavelength of 538 nm. Cytolytic activity was determined as a percentage of lysis and calculated using the following equation: Lysis (%) = (E - S) / (M - S) × 100, where E is the fluorescence measured in the co-cultures of target and effector cells, S is the spontaneous fluorescence of the target cells, and M is the maximum fluorescence measured after lysis of all cells with a buffer containing 0.5% Triton X-100, 10 mM Tris-HCl (pH 7.4) and 10 mM EDTA.

#### CDC

CDC by EpCAM was assayed as follows: Caco-2 cells were labeled with 10 µg/ml Calcein-AM and resuspended in DMEM with 10% FBS. Caco-2 cells were plated in 96-well plates at 2×10^4^ cells/well, and 10% Low-Tox-M rabbit complement (Cedarlane Laboratories) with 100 µg/ml anti-EpCAM mAb or control mouse IgG_2a_ was added to each well. Following a 4-h incubation at 37°C, Calcein-AM release into the supernatant was determined in each well. Fluorescence intensity was calculated as described in the ADCC section.

#### Antitumor activity of anti-EpCAM mAb in colorectal adenocarcinoma xenografts

A total of 32 5-week-old female BALB/c nude mice were purchased from Charles River Laboratories, Inc. After a 2-week acclimation period, the mice were used in experiments at 7 weeks of age. Caco-2 cells (0.3 ml; 1.33×10^8^ cells/ml in DMEM) were mixed with 0.5 ml BD Matrigel Matrix Growth Factor Reduced (BD Biosciences), and 100 µl of this suspension (5×10^6^ cells) was injected subcutaneously into the left flank of each animal. On day 1 post-inoculation, 100 µg anti-EpCAM mAb or control mouse IgG in 100 µl PBS was injected i.p. Additional antibody inoculations were performed on days 7 and 12. Tumor formation was measured in mice in the treatment and control groups on days 7, 8, 12, 15 and 17 after Caco-2 cell injection. On day 17 after cell implantation, all mice were euthanized by cervical dislocation, and tumor diameters and volumes were measured and recorded.

#### Statistical analyses

Data are presented as the mean ± SEM. Statistical analysis was conducted with one-way ANOVA and Tukey's multiple comparisons tests for ADCC and CDC; one-way ANOVA and Sidak's multiple comparisons tests for tumor volume and mouse weight; and Welch's t-test for tumor weight. All calculations were performed with GraphPad Prism 7 (GraphPad Software, Inc.). P<0.05 was considered to indicate a statistically significant difference.

## Results

### 

#### Establishment and characterization of the anti-EpCAM mAb

The anti-EpCAM mAb was established by immunizing one mouse with CHO/EpCAM cells and fusing its spleen cells with P3U1 cells. Supernatants from hybridomas, which were positive for CHO/EpCAM and negative for CHO-K1, were selected by flow cytometry. Further screening by immunohistochemistry and western blotting was performed for validation, resulting in the establishment of EpMab-16 (IgG_2a_, κ).

**Figure 1.**
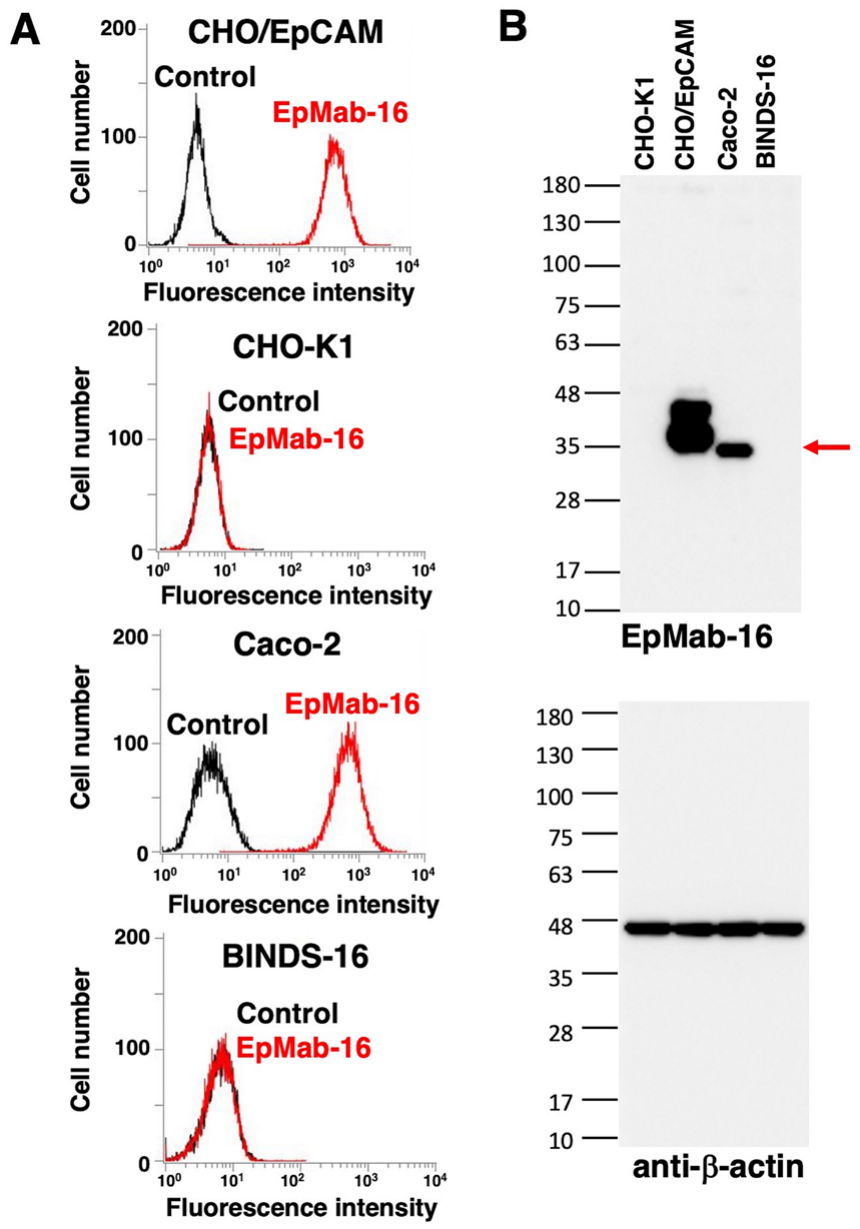
Recognition of EpCAM by EpMab-16. (A) Flow cytometry analysis using EpMab-16. CHO/EpCAM, CHO-K1, Caco-2 and BINDS-16 cells were treated with EpMab-16 (1 µg/ml) or buffer control, followed by secondary antibodies. (B) Western blot analysis of EpCAM expression using EpMab-16. Red arrow denotes 35-kDa EpCAM. EpCAM, epithelial cell adhesion molecule; EpMab-16, anti-EpCAM monoclonal antibody; BINDS-16, Caco-2/EpCAM-knockout.

Flow cytometry was performed to assess the sensitivity of EpMab-16 in CHO/EpCAM cells and the Caco-2 colorectal adenocarcinoma cell line. As presented in Fig. 1A, EpMab-16 bound to CHO/EpCAM cells, but not CHO-K1 cells. EpMab-16 also bound to Caco-2 but not BINDS-16 cells, indicating that EpMab-16 was specific for EpCAM in the colorectal adenocarcinoma cell line.

Western blotting was performed to further assess the sensitivity of EpMab-16. Lysates of CHO-K1, CHO/EpCAM, Caco-2 and BINDS-16 cells were probed. As demonstrated in Fig. 1B, EpMab-16 detected the 35-kDa band of EpCAM in lysates from CHO/EpCAM and Caco-2 cells, whereas this band was not present in lysates from CHO-K1 and BINDS-16 cells, indicating that EpMab-16 specifically detected both exogenous and endogenous EpCAM. The molecular weight of EpCAM between CHO/EpCAM and Caco-2 was different as a PA tag (12 amino acids) was added to C-terminus of EpCAM in CHO/EpCAM cells.

**Figure 2.**
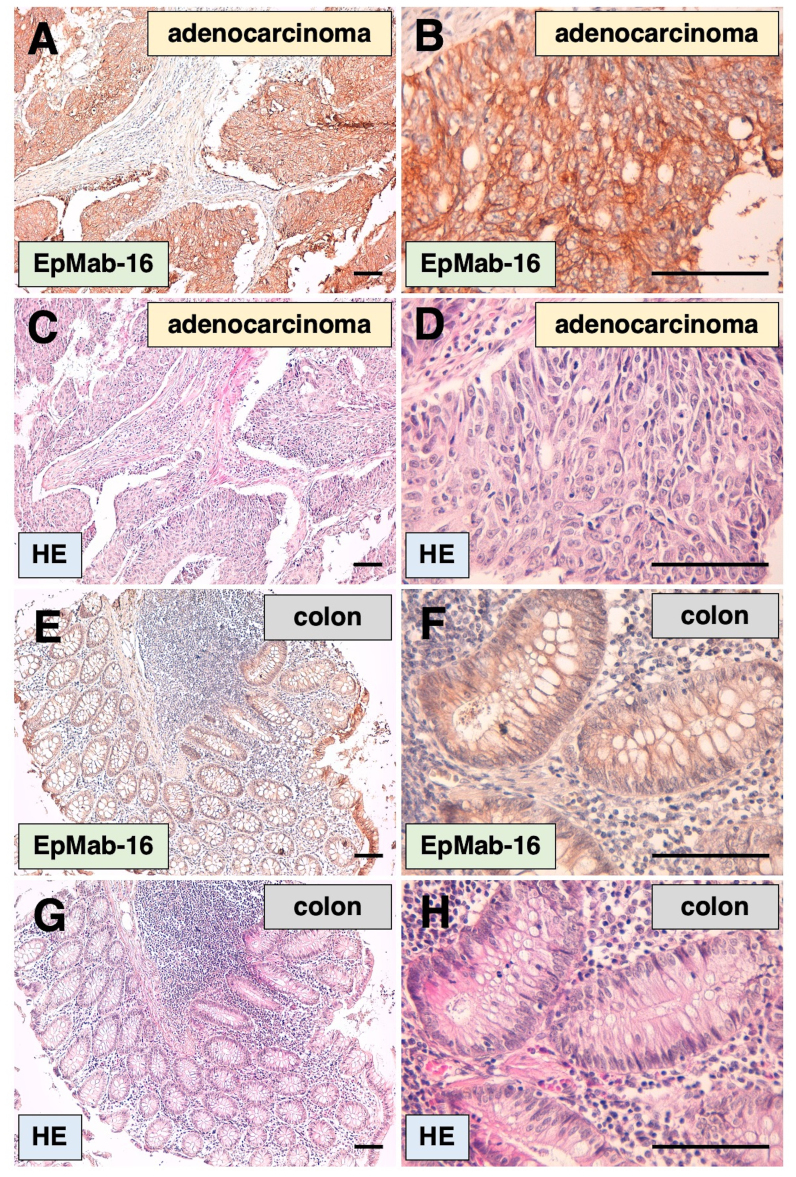
Immunohistochemical analysis of colorectal adenocarcinoma and normal colon tissues using EpMab-16. (A and B) FFPE tissue sections of colorectal adenocarcinoma stained with 1 µg/ml EpMab-16. B is a magnified version of A. (C and D) HE staining of consecutive colorectal adenocarcinoma tissue sections. D is a magnified version of C. (E and F) FFPE tissue sections of normal colon stained with 1 µg/ml EpMab-16. F is a magnified version of E. (G and H) HE staining against consecutive normal colon tissue sections. H is a magnified version of G. Scale bar, 100 µm. EpMab-16, anti-epithelial cell adhesion molecule monoclonal antibody; FFPE, formalin-fixed paraffin-embedded; HE, hematoxylin and eosin.

EpMab-16 detected membrane antigens in colorectal adenocarcinoma tissues in the immunohistochemical analysis (Fig. 2A and B). Among six cases of colorectal adenocarcinoma, five (83%) were positively stained by EpMab-16. No staining was observed without the primary mAb (data not shown). H&E staining was performed to stain nucleus as blue and cytosol as pink, using consecutive colorectal adenocarcinoma tissue sections (Fig. 2C and D). Furthermore, EpMab-16 weakly detected membrane antigens in normal colon tissues (Fig. 2E and F). Among six samples of normal colon tissues, three tissues (50%) were stained by EpMab-16. No staining was observed without the primary mAb (data not shown). H&E staining was performed to stain nucleus as blue and cytosol as pink, using consecutive normal colon tissue sections (Fig. 2G and H).

**Figure 3.**
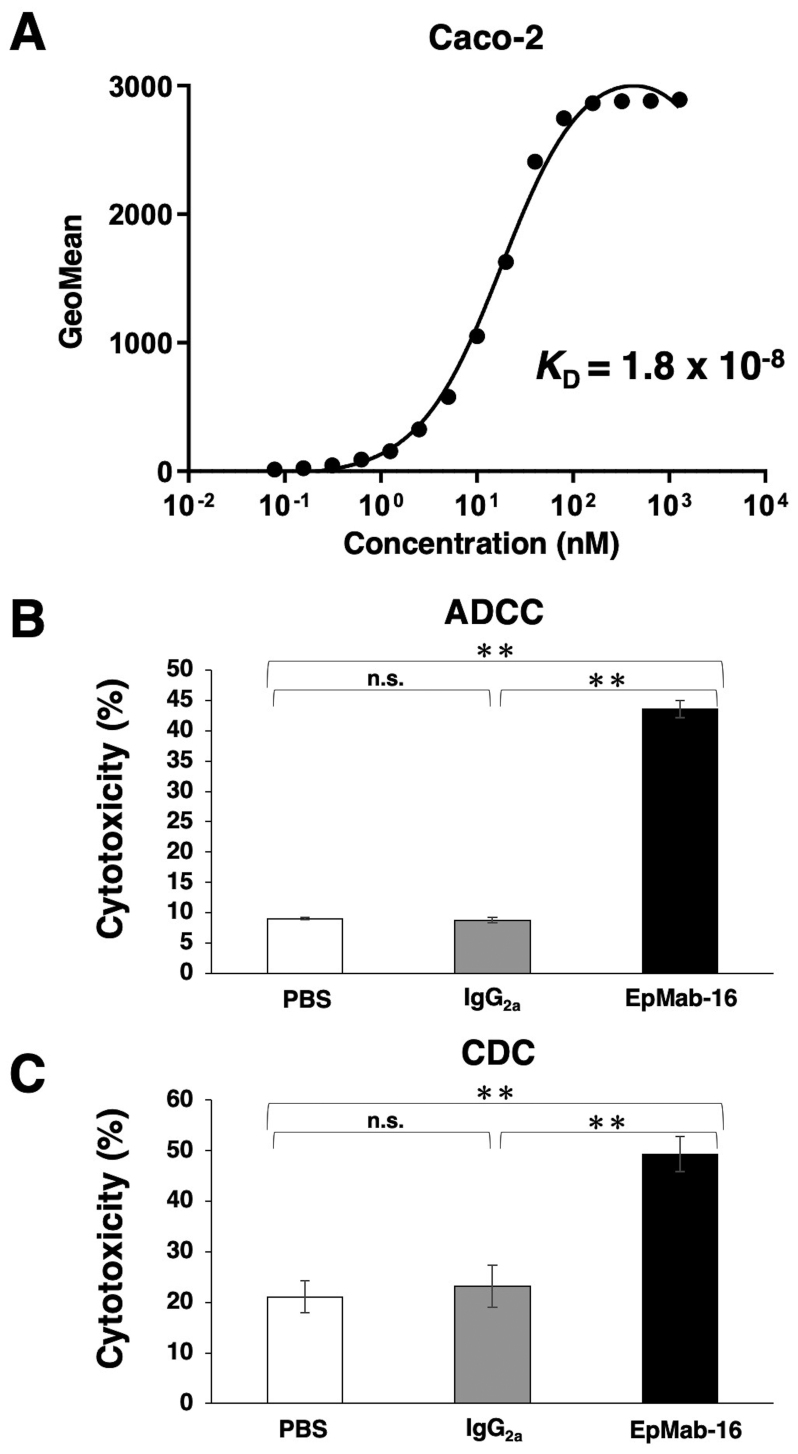
Binding affinity, ADCC and CDC of EpMab-16. (A) Determination of binding affinity of EpMab-16 in Caco-2 cells. (B) ADCC activity of EpMab-16, control mouse IgG_2a_ and control PBS in Caco-2 cells. (C) CDC activity of EpMab-16, control mouse IgG_2a_ and control PBS in Caco-2 cells. **P<0.01; n.s., not significant. EpMab-16, anti-epithelial cell adhesion molecule monoclonal antibody; ADCC, antibody-dependent cellular cytotoxicity; CDC, complement-dependent cytotoxicity; *K*_D_, dissociation constant.

Kinetic analysis of the interactions of EpMab-16 with Caco-2 cells was subsequently analyzed using flow cytometry. The *K*_D_ for EpMab-16 in Caco-2 cells was calculated to be 1.8×10^−8^ M (Fig. 3A), indicating a moderate binding affinity of EpMab-16 to colorectal adenocarcinoma cells.

#### ADCC and CDC activities of EpMab-16 in a colorectal adenocarcinoma cell line

The present study further investigated whether EpMab-16 induced ADCC and CDC antitumor activity in an EpCAM-expressing Caco-2 colorectal adenocarcinoma cell line. As presented in Fig. 3B, EpMab-16 exhibited higher ADCC (44% cytotoxicity) in Caco-2 cells compared with that of control mouse IgG_2a_ (8.7% cytotoxicity; P<0.01) or control PBS (9.0% cytotoxicity; P<0.01) treatment. EpMab-16 was also associated with more robust CDC activity (49% cytotoxicity) in Caco-2 cells compared with that of control mouse IgG_2a_ (23% cytotoxicity; P<0.01) or control PBS (21% cytotoxicity; P<0.01) treatment (Fig. 3C). These results suggested that EpMab-16 induced strong ADCC and CDC antitumor activity *in vitro*.

#### EpMab-16 antitumor activity in mouse xenografts of Caco-2 colorectal adenocarcinoma cells

**Figure 4.**
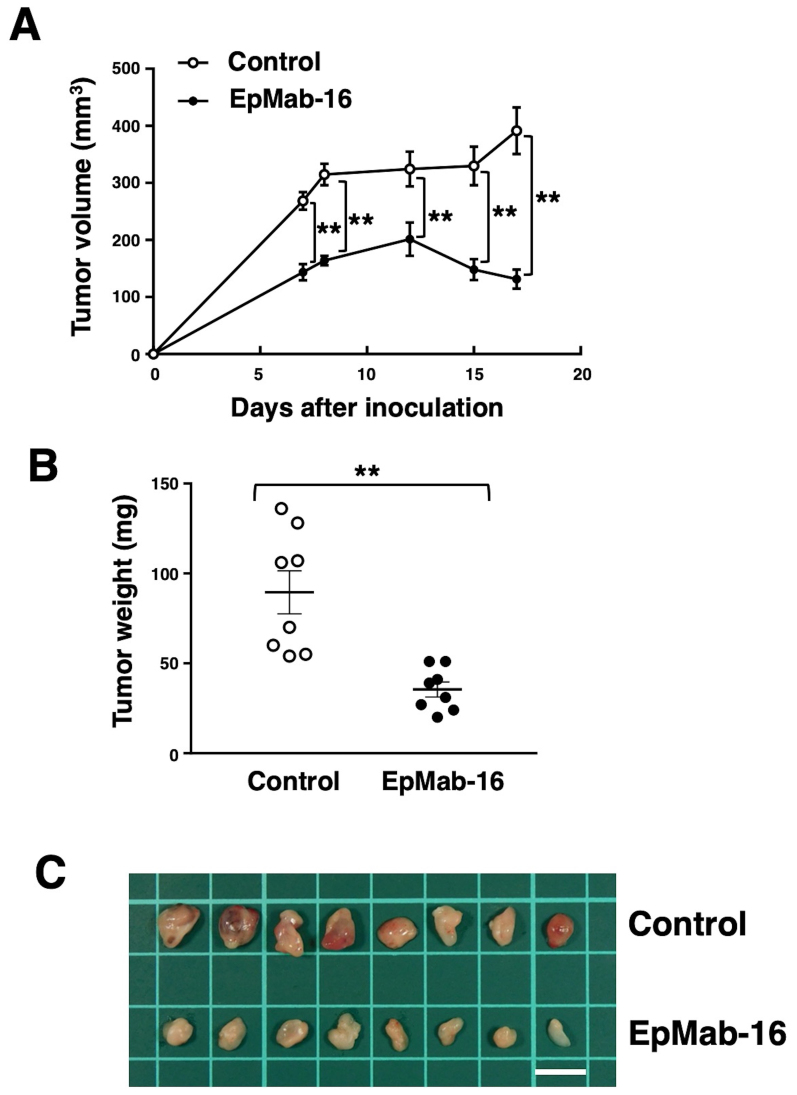
Antitumor activity of EpMab-16 in Caco-2 ×enografts. (A) Tumor volumes of Caco-2 ×enografts. Caco-2 cells were injected subcutaneously, and 100 µg EpMab-16 or control mouse IgG was injected into the treatment and control mice, respectively; additional antibodies were injected on days 7 and 12. Tumor volume was measured on days 7, 8, 12, 15 and 17. (B) Tumor weights of Caco-2 ×enografts resected from the EpMab-16 and control mouse IgG groups on day 17. (C) Resected tumors of Caco-2 ×enografts from the EpMab-16 and control mouse IgG groups on day 17. Scale bar, 1 cm. **P<0.01. EpMab-16, anti-epithelial cell adhesion molecule monoclonal antibody.

We further examined whether EMab-16 exerts antitumor activity *in vivo*. On days 1, 7 and 12 after Caco-2 cell injections into the mice, the Caco-2 ×enograft mouse models were injected with EpMab-16 or control mouse IgG. During the animal experiment, no apparent weight loss due to tumor burden or organ failure was observed among the mice. EpMab-16-treated mice exhibited significantly lower tumor growth on days 7, 8, 12, 15 and 17 (all P<0.01) compared with that in IgG-treated control mice (Fig. 4A). Tumor volume reduction by EpMab-16 treatment reached 66% relative to the control group on day 17. Tumors from EpMab-16-treated mice weighed significantly less compared with those from IgG-treated control mice (60% reduction; P<0.01; Fig. 4B). Resected tumors on day 17 are depicted in Fig. 4C. Total body weights did not significantly differ between the treatment and control groups (Fig. S1). These results indicated that EpMab-16 reduced the growth of Caco-2 ×enografts, but did not altogether eliminate it.

## Discussion

The present study aimed to determine whether a novel anti-EpCAM mAb may be useful for treating colorectal adenocarcinoma. First, a sensitive and specific anti-EpCAM mAb, EpMab-16, was developed, which exhibited high reactivity for colorectal adenocarcinoma by flow cytometry, western blotting and immunohistochemical analyses. Notably, the results also suggested that EpMab-16 exhibited diagnostic efficacy in FFPE tissues as pathological diagnosis utilizes FFPE tissues. EpMab-16 was demonstrated to possess strong ADCC and CDC activity against the Caco-2 colorectal adenocarcinoma cell line *in vitro*, and significantly reduced not only the volume but also the weight of Caco-2 ×enografts *in vivo*. Although tumor reduction was determined to be 66% by volume and 60% by weight on day 17, the reduction was not sufficient to eliminate the tumor entirely.

Cancer stem cells (CSCs) exhibit specific characteristics including decontrolled self-renewal, tumor-initiating and tumor-promoting properties, and chemotherapy resistance. Therefore, targeting CSCs is thought to be a promising approach to treat cancer (22). Since CD133 is thought to be one of CSC markers for CRC (23–25), we previously developed an anti-CD133 mAb (CMab-43, mouse IgG_2a_) (17) and investigated whether CD133 may represent a therapeutic target in colorectal adenocarcinoma using CMab-43 (26). Importantly, CMab-43 exerted antitumor activity in Caco-2 ×enograft models at a dose of 100 µg/mouse/week administered three times, suggesting that CMab-43 may be useful for antibody therapy against CD133-positive CRC (26). In the present study, the effects of an anti-EpCAM mAb, EpMab-16, were examined *in vitro* and *in vivo*, as EpCAM is also known to be a CSC marker for CRC (25). In the present study, EpMab-16 also showed antitumor activity against CRC in a murine xenograft model. CSC marker-expressing cells may be heterogenous; therefore, the combination of mAbs or anticancer drugs to kill the CSC marker-expressing cancer cells could be an ideal strategy to treat CRC.

In another recent study, we established an anti-HER2 mAb (H_2_Mab-19, mouse IgG_2b_) (27). Our previous study demonstrated that H_2_Mab-19 significantly reduced tumor development in HER2-expressing breast cancer cells (BT-474), oral cancer cells (HSC-2 and SAS) and CRC cells (Caco-2) xenograft models, suggesting that treatment with H_2_Mab-19 may be a useful therapy for patients with HER2-expressing cancers (27,28). Although H_2_Mab-19 showed significant antitumor activity, immunohistochemical analysis revealed that HER2 expression was diminished in the remaining cancer cells after H_2_Mab-19 treatment (28). These results suggest that H_2_Mab-19 treatment may not be fully effective for patients with HER2-expressing cancers. In the current study, an EpCAM-targeting mAb, EpMab-16, was developed. To enhance the therapeutic effects *in vivo*, the combinational use of different mAbs, such as H_2_Mab-19 and EpMab-16 may be required to treat HER2 and/or EpCAM-expressing CRC cells.

The success of antibody-based therapeutics of cancer depends on the target antigen and the antibody to be used. It has been reported that EpCAM is upregulated in 94% of CRCs (29), whereas EpCAM expression is restricted to only the basolateral membrane of epithelial cells in normal tissues, and therapeutic agents have limited access to it (30), underscoring the importance of EpCAM as a therapeutic target. Since EpMab-16 appears to react with not only the basolateral membrane, but also the apical membrane of epithelial cells according to the results of the present study, the expression pattern of EpCAM should be further investigated by comparing EpMab-16 with other anti-EpCAM mAbs.

A number of antibody-based therapeutic approaches targeting EpCAM have been developed (10). Previously, Liao *et al* (31) generated an anti-EpCAM mAb EpAb2-6. Using a colon cancer xenograft model, they demonstrated that EpAb2-6 induced CRC cell death by inhibiting EpCAM signaling rather than by acting through the ADCC or CDC (31). On the other hand, EpMab-16 generated in the present study possessed high ADCC and CDC activities against CRC cells and exhibited antitumor activity *in vivo*. Another previous study demonstrated that an anti-EpCAM toxin-conjugated antibody Oportuzumab monatox (also termed VB4-845) was effective against squamous cell carcinomas of the head and neck and non-muscle invasive bladder cancer, and well-tolerated in clinical trials (phase I and II) (32). Considering that EpCAM is also expressed in tumor-initiating cells, antibody-based therapies against EpCAM may kill not only proliferating cancer cells, but also drug-resistant dormant CRC cells (6). These results suggest that the EpCAM-targeted immunotherapy may be a promising therapeutic strategy for CRC. To further develop EpCAM-targeted cancer therapy, further studies are required to elucidate the precise role of EpCAM inhibition among various cancer cells.

## Supplementary Material

Supporting Data

## Data Availability

The datasets used and/or analyzed during the study are available from the corresponding author on reasonable request.

## Abbreviations

ADCC: antibody-dependent cellular cytotoxicity
CBIS: cell-based immunization and screening
CDC: complement-dependent cytotoxicity
EpCAM: epithelial cell adhesion molecule
mAb: monoclonal antibody
