# Supplementary material for: The anti-epithelial cell adhesion molecule (EpCAM) monoclonal antibody EpMab-16 exerts antitumor activity in a mouse model of colorectal adenocarcinoma
Source: Oncol Lett. 2020 Oct 23;20(6):383. doi: 10.3892/ol.2020.12246 (PMC7608076; doi:10.3892/ol.2020.12246)

Figure S1. Body appearance and body weights of mice implanted with Caco-2 xenografts. (A) Mouse appearance on day 17. Scale bar, 1 cm. (B) Mouse weight was measured on days 0, 1, 5, 7, 12, 15 and 17. n.s., not significant. EpMab16, epithelial cell adhesion molecule monoclonal antibody.

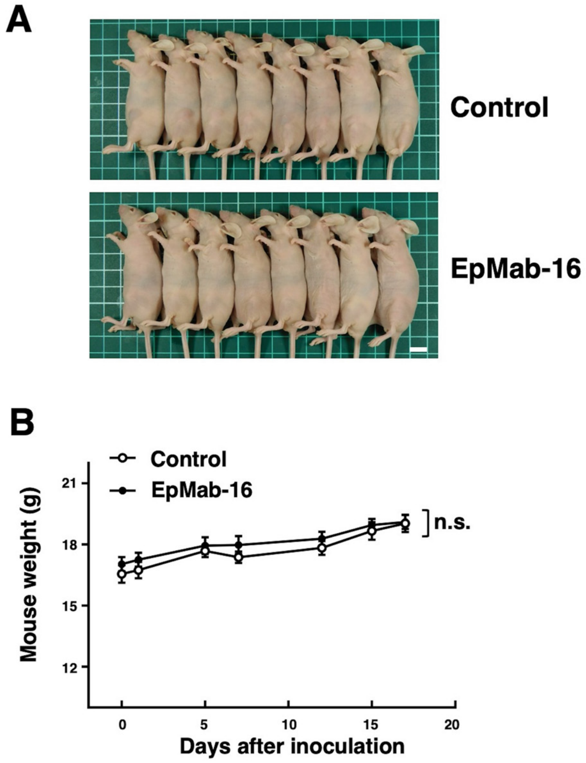

Supplement: Supporting Data [file Supplementary_Data.pdf]
